# Protocol for an app-based affective control training for adolescents: proof-of-principle double-blind randomized controlled trial

**DOI:** 10.12688/wellcomeopenres.15229.2

**Published:** 2019-10-02

**Authors:** Susanne Schweizer, Jovita T. Leung, Rogier Kievit, Maarten Speekenbrink, William Trender, Adam Hampshire, Sarah-Jayne Blakemore

**Affiliations:** 1 Institute of Cognitive Neuroscience, University College London, London, UK; 2 MRC Cognition and Brain Sciences Unit, University of Cambridge, Cambridge, UK; 3 Department of Experimental Psychology, University College London, London, UK; 4 Department of Medicine & Centre for Neurotechnology Computational, Cognitive & Clinical Neuroimaging Laboratory, Imperial Collge London, London, UK

**Keywords:** Mental health, Adolescence, Emotion regulation, Affective control, App-based training

## Abstract

**Background: **75% of all mental health problems have their onset before the end of adolescence. Therefore, adolescence may be a particularly sensitive time period for preventing mental health problems. Affective control, the capacity to engage with goal relevant and inhibit distracting information in affective contexts, has been proposed as a potential target for prevention. In this study, we will explore the impact of improving adolescents’ affective control capacity on their mental health.

**Methods: **The proof-of-principle double-blind randomized controlled trial will compare the effectiveness of an app-based affective control training (AffeCT) to a placebo training (P-Training) app. In total, 200 (~50% females) adolescents (11-19 years) will train for 14 days on their training app. The AffeCT will include three different *n*-back tasks: visuospatial, auditory and dual (i.e., including both modalities). These tasks require participants to flexibly engage and disengage with affective and neutral stimuli (i.e., faces and words). The P-Training will present participants with a perceptual matching task. The three versions of the P-Training tasks vary in the stimuli included (i.e., shapes, words and faces). The two training groups will be compared on gains in affective control, mental health, emotion regulation and self-regulation, immediately after training, one month and one year after training.

**Discussion: **If, as predicted, the proposed study finds that AffeCT successfully improves affective control in adolescents, there would be significant potential benefits to adolescent mental health. As a free app, the training would also be scalable and easy to disseminate across a wide range of settings.

**Trial registration: **The trial was registered on December 10th 2018 with the International Standard Randomised Controlled Trial Number (Registration number: ISRCTN17213032).

## Abbreviations

AffeCT = Affective control training; P-Training = Placebo training; DERS = Difficulties in Emotion Regulation Scale

## Background

75% of all mental health problems have their onset before the end of adolescence ^1^. Many of these disorders, for example major depressive disorder, will be recurrent throughout the lifespan creating large costs in human suffering ^2– 4^. Adolescence – here defined as starting with puberty and ending with the attainment of an independent adult role (10–24 years ^5^) – thus may be a particularly sensitive time period for prevention of mental health problems ^6^. In this study, we will explore the impact of improving adolescents’ affective control capacity on their mental health.

Affective control, the capacity to flexibly engage and disengage from affective information as required by current goal-demands, is impaired across a wide range of mental health problems ^7^. Poor affective control capacity has been shown to be associated with poor mental health outcomes over and above neutral ‘cool’ cognitive control during adolescence ^8– 12^. We have previously suggested that this association between affective control and mental health can be partially accounted for by emotion regulation ^12^. That is, affective control constitutes the cognitive building blocks of successful emotion regulation. Emotion regulation refers to the automatic and volitional processes deployed to modify an individual’s affective experiences ^13^. Improving affective control in adolescents, whose everyday environments can include high levels of negative affect and affective fluctuations ^14– 17^, may then confer benefits to emotion regulation capacity and mental health.

Studies conducted in adults have shown that training affective control leads to improvements in both emotion regulation capacity and self-reported mental health ^18– 21^. Cool cognitive control training has also been shown to be effective in reducing symptoms of depression and anxiety, as well as improving emotion regulation ^22– 27^.

The cognitive training literature in children and adolescents has largely focused on remediation for learning difficulties as well as neurodevelopmental disorders such as attention deficit and hyperactivity disorder (ADHD ^28, 29^). Less is known about the impact of cognitive control training on young people’s mental health ^30^. A notable exception is the literature on cognitive training for adolescents with psychotic symptoms ^31, 32^, which synthesizing evidence suggests shows promising effects on symptoms and functioning ^33^. However, given that affective control in particular may be impaired in adolescents with high levels of mental health problems, we will trial the effect of an affective control training (AffeCT) paradigm in our forthcoming study. Specifically, we will explore whether AffeCT improves adolescents’ mental health and emotion regulation capacity and whether the magnitude of these improvements differs as a function of age.

### The present study

To investigate the potential of AffeCT in adolescents, the current study will include 200 adolescents (11–19 years). Including this age range will allow us to investigate the potential age-related differences in the effectiveness of training, as shown in studies using cool cognitive training paradigms ^34^.

The training that will be used in the current study is a variant of a paradigm we applied successfully in adults and a preliminary study in adolescents with posttraumatic stress disorder ^21, 35, 36^. The impact of the AffeCT will be assessed in a proof-of-principle double-blind randomized controlled trial. In the AffeCT, participants will be presented with visuospatial, auditory and dual (combined visuospatial and auditory) versions of the *n*-back task. The three versions will, respectively, require participants to continuously update faces or words or both. On the first three days of the 14-day training programme, participants will train on one version each day. On days 4–14, participants are free to select any or all of the training versions. However, both training groups will be provided with a rationale suggesting that training on version C is likely to confer more benefits than the other two versions. By providing a rationale for one training being associated with superior benefits compared to others, participants should be motivated to select C over the other two training versions. However, C is more cognitively demanding, therefore requiring self-regulation to engage in this task over the others for potential future benefits. We hypothesise that opting to engage in a more challenging but potentially more beneficial task is an index of self-regulation, which has been shown to be associated with mental health across the lifespan ^37^. This will allow us to explore the role of self-regulation in cognitive training and any effects on mental health outcomes.

The tasks train affective control by requiring effective engagement and disengagement with affective information depending on task-demands. Affective valence is introduced to the training by including valenced stimuli. Specifically, the AffeCT will include 20% neutral, 20% positive and 60% negative stimuli. The rationale for including stimuli of different valences is that mental health problems can be characterised by difficulties disengaging from negative material ^38^, avoidance of negative (e.g., threatening) information ^39^ or aberrant processing of positive information ^40^. Moreover, we have recently shown in a meta-analysis that, individuals with mental health problems, affective control, measured with working memory tasks such as the training task, is similar across positive and negative valence ^7^.

The effectiveness of the AffeCT will be compared with an active placebo training (P-Training), which includes three versions: shapes, words and faces from a feature match task. The P-Training requires participants to indicate whether the items presented in two panels are matched or mismatched. The training was designed to be minimally demanding on cognitive control, while exposing participants to the same stimuli as the AffeCT.

To investigate potential benefits of the AffeCT on mental health or emotion regulation, participants will complete self-report measures immediately after training, after one month, and after one year. Additionally, the three facets of affective control – *inhibiting* attention and responses toward goal-irrelevant affective information, *updating* affective information or updating information in the context of affective distraction, and *shifting* flexibly between affective and non-affective task demands – will be assessed using experimental paradigms. Inhibition will be assessed with a modified version of Preston and Stansfield’s ^41^ affective Stroop task, which requires participants to categorize adjectives as either happy or sad that are superimposed over task-irrelevant faces that are either congruent, incongruent or neutral (i.e., scrambled). Updating will be assessed with the affective digit backward span task. In this task digits are serially presented over either a neutral or affective background image and then recalled in reverse order (modified version of standard digit span task; 42). Finally, shifting will be assessed with an affective card sorting task, which requires participants to flexibly switch between affective and neutral sorting rules ^12^.

This study will allow us to investigate the following four hypotheses:

1. Affective control can be improved in adolescents ( *affective control training hypothesis*). To investigate this hypothesis, we will compare individuals’ performances on the affective *n*-back task across the two training groups.
2. AffeCT compared to P-Training will lead to greater improvements in all facets of affective control as measured by non-trained affective control tasks, including affective inhibition, updating and shifting tasks ( *affective control facets hypothesis*).
3. The benefits of AffeCT will vary as a function of age ( *age-related change hypothesis*).
4. Increases in affective control from pre- to post-training will be associated with fewer self-reported mental health problems and emotion regulation difficulties, as well as higher levels of self-reported self-control, at each assessment time point ( *mental health hypothesis*).

## Methods

### Study setting

The study will be run in schools in London, Cambridge and surrounding areas, and at the UCL Institute of Cognitive Neuroscience, UK.

### Participants

In total, 200 adolescents (~50% female, 11–19 years) will be recruited through schools, advertisements on the lab website, the MQ research portal, the Anna Freud Centre “Schools in Mind” website, and social media. Recruitment will be stratified by age to ensure a proportional representation of each chronological year group. Including 200 participants (100 per training group) results in ≥ 93% power to detect an effect on our first hypothesis that affective control can be trained in adolescence. Power was established with time as within-subjects factor, training group as fixed factor and participants as random factors. The effect size for the interaction was estimated as small to medium *d* = .40, based on our previous training studies in adolescents ^36^ and adults ^21, 35^. The calculator used was https://jakewestfall.shinyapps.io/pangea/. 

***Eligibility criteria.*** To be included participants will have to be between 11–19 years old and speak English fluently. Participants will be excluded from the study if they have a history of traumatic head injury, a diagnosed neurological or neurodevelopmental disorder, or if they are currently enrolled in another cognitive training intervention.

***Allocation procedure.*** Included participants will be randomized to either the AffeCT or the P-Training groups. Condition allocation will be concealed to experimental staff by using computer-generated condition assignment (using Sealed Envelope simple randomisation service) stratified by age (young adolescents 11–14 years and mid-late adolescents 15–19 years; in line with: 12). Allocation will be based on a blocked randomization sequence with randomly mixed block sizes (2–6), which prevents the experimenter from deducing any potential sequencing even with awareness of the randomization type ^43^. One experimenter (SS) will only conduct pre-training assessments and not be involved with any further participant testing as they will answer any queries about the training and technical issues that the participants may face during the training.

***Blinding.*** For blinding procedures see *extended data* ^44^. The procedures are uploaded to the trial registration page as a time-stamped private document and will be made available online upon study completion. Following the final participant’s follow-up assessment (one year after the second testing session, T2), all participants will receive an email describing the study purpose and giving them access to all training tasks for 12 months.

### Training procedure and timeline

**Figure 1.**
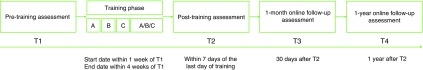
Study timeline. T1 – T4 = Assessment time point 1 – 4; A/B/C = refers to the three different versions of the training tasks available in both training groups; Pre- and post-training assessment = Assessment sessions run prior to and after completing the training phase; Training phase = period of training on the app; 1-month and 1-year online follow up assessment = key outcomes will be assessed online one month and one year following the completion of the training.

Participants complete a pre-training assessment, followed by 14 days of training within a four-week period. The training will be completed individually by the participants on their own devices (any device that supports mobile apps). Within one week of the end of the four-week period, participants will complete the post-training assessment. Any deviations from the per protocol timeline due to the constraints of school-based testing will be accounted for in our analyses (see *Statistical analyses* section). Thirty days after the post-training assessment, participants will be asked to complete an online follow-up assessment. A final follow-up assessment will be completed one year after the post-training session. For a schematic overview of the study timeline see Figure 1.

***Training phase.*** During the training phase, participants from both training groups will be presented with three different training tasks (see below for descriptions). On the first three days they will complete a different version of the training task each day. The presentation order of the three versions will be fixed for days 1–3. The P-Training group will complete the shapes (A), words (B), and faces (C) versions on the first, second, and third day of training, respectively. The AffeCT group will complete the visuospatial (A), auditory (B), and dual (i.e., including both modalities; C) versions of the training task on the first three days of training. From the fourth day of training onward participants in both groups will be free to select any of the three different versions of their training schedule.

At the beginning of the training, both groups will be told that they should spend as much time as possible training on version C due to its benefits to attention, memory and emotion regulation. Version C in the AffeCT will be significantly more cognitively demanding ^45^ than A and B, whereas there are no differences in cognitive demands between versions A, B or C in the P-Training. Emulating the design of established measures of academic diligence and self-regulation ^46– 48^, the ratio of time spent training on version C relative to A and B will be taken as a behavioural index of self-regulation.

***Procedure on each training day.*** On each training day tasks will be populated with a different set of stimuli. In both training groups participants will be given the option to end the training any time from 10 mins onward. The full training session will take between 20–30 mins depending on the level achieved. There will be no limit on the number of training sessions they can complete during a day. Training sessions that are less than 10 mins will not be considered as full training sessions, and will not be included in the analyses, nor will participants be compensated for these sessions.

Each time they start the training, participants will be asked four brief questions about their mood, affect regulatory intentions, social context and current activity. To assess current mood, participants will be asked, “How happy do you feel right now?”. They will provide their mood rating by moving the cursor on a visual analogue scale ranging from “Very unhappy” to “Very happy”. Affect regulatory intentions will be assessed with the question, “Are you trying to change the way you feel right now?”. They will be offered nine answer options from a dropdown menu. Participants will be able to select “No.” or “Yes, by …” followed by different types of regulatory strategies (i.e., distraction, problem-solving, behavioural activation, reappraisal, avoidance, social support, acceptance or other). Social context will be assessed by selecting from a dropdown menu to indicate whether right now they are: “Alone”, “With others (friends/family)”, or “With others (strangers).” Finally, participants will indicate their current activity from a selection of eleven options on a dropdown menu (e.g., commuting, school/work).

***Adherence/retention.*** Participants will be compensated for each section of the study to incentivise enrolment and study completion. They will be paid £10 for each pre- and post-training assessments (T1 & T2) and £5 for both the online follow-up assessments (T3 & T4). Participants will additionally receive £2 per completed training day. If participants complete two or more sessions on a single day they will be paid £5.

*Training.* In addition to payments, retention will be optimised by sending participants a daily training reminder at 8am. Participants who have not completed at least 10 mins of training by 5pm will be sent an additional reminder, informing them that they have a training session waiting for them. A final reminder will be sent at 8pm for any participants who have not completed their minimum training requirement by then.

*Follow-up.* Two weeks after the initial request to complete the follow-up assessments, email reminders will be sent to incentivise follow-up completion. Reminder emails will be sent at weekly intervals, until the follow-up assessments are completed or until the maximum number of reminders (i.e., three) has been sent, whichever comes first.

### Training tasks

**Figure 2.**
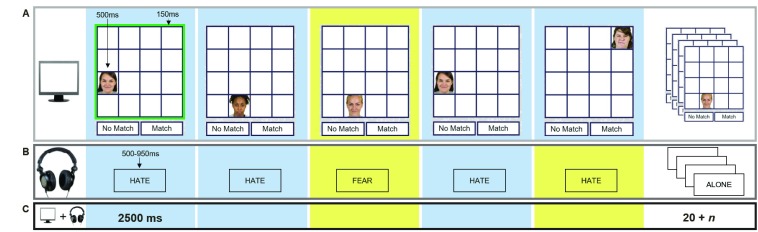
Affective control training tasks. The figure depicts sample trials for each of the three training tasks: **A**) visuospatial *n*-back, **B**) auditory *n* –back, and **C**) dual *n*-back task. Trials depicted with a light blue background require a “No Match” button press, whereas yellow backgrounds indicate “Match” (i.e., target) trials in the respective modality. The green border provides feedback to participants, where green indicates the response was correct, whereas a red border appears for incorrect trials. Feedback is provided after each response or when a trial times out. The example block in Figure 2 is depicted for *n* = 1. Match trials for the visuospatial *n*-back training task are trials where the current face is presented in the same location as the face *n* positions back. For auditory *n*-back match trials, the same word is presented as the one *n* trials back. The dual *n*-back training task includes both modalities and both types of target trials (for additional buttons appearing on screen with the dual *n*-back see the task description below). 2500ms = the maximal (duration is self-paced up to 2500ms) time between onset of one stimulus and the next (i.e., total trial time); 500ms = face presentation time; 150ms = feedback presentation time; 500-950ms = word presentation time. 20 + *n* = each block consists of 20 + *n* trials.

***Affective control training.*** The three versions of the AffeCT tasks are described below and depicted in Figure 2.

***Training progression.*** The first three days will start at *n* = 1. From day 4 onward participants will select one of the versions to train on and training will start at the average level of *n*-back achieved on the previous training session with the selected version. During each individual training session, the difficulty level will be titrated to each participant’s maximum capacity with *n* increasing by 1 if performance reaches ≥ 70% accuracy and decreasing by 1 when accuracy is ≤ 30%. Accuracy feedback will be provided after each response. A red boarder will flash up around the grid for false alarms (participant presses Match on a non-target trial) or misses (participant presses No Match on a target trial or fails to provide any response). A green boarder will flash up for all correctly classified trials.

***Stimuli.*** Each of the training versions will include 20% neutral stimuli and 80% affective stimuli to train the flexible engagement and disengagement from affective information. 30% of the trials will constitute target trials. The words included in the dual and auditory versions of the AffeCT are derived from the Affective Norms for English Words database ^49^ and with the exception of the positive words were included in previous versions of this training task ^21, 35^. Positive stimuli are included in the current training task because of the salience of positive material in adolescence ^50^, as well as research showing a critical role of reward processing in the onset of mental health problems ^51, 52^. The words are 20% neutral, 30% positive and 50% negative.

The faces stimuli were selected from several different databases, which are licenced for use online, to provide a diverse stimulus set in terms of demographics and emotional expressions. The databases included are: the Chicago Face Database ^53^, the Radboud Faces Database ^54^, the London Face Research Set ^55^, the Emotional Faces Stimulus Set ^56^, and the NIMH Child Emotional Faces Picture Set ^57^. Our final stimulus set includes child, adolescent and adult faces of female and male gender. The ethnic appearances of the faces included are African, Asian, Caucasian, Latin American, Mediterranean, Middle Eastern, and Mixed Race. We denote the ethnicity here as appearance only as not all the actors’ origins were recorded across the different databases. The emotional expression of the faces included are happy, angry, fearful, sad, and neutral. In each training session 50% of the faces are female, 50% of the faces are from child and adolescent models. The affective expressions included in each training session are 20% neutral, 20% angry, 20% fearful, 20% sad (i.e., 60% negative), 20% happy.

***Visuospatial n-back.*** In the visuospatial *n*-back task faces appear for 500 ms on a 4×4 grid. The task requires participants to indicate within 2.5 s whether the face they are seeing in the current trial is presented in the same location as the face presented *n* trials back. Responses are provided via “No Match” or “Match” button press.

***Auditory n-back.*** In the auditory version of the training task participants are presented with words over headphones. On each trial they have 2.5 s to indicate via button press (see visuospatial *n*-back), whether the word presented in the current trial is the same as they heard *n* trials back.

***Dual n-back.*** The dual version of the task presents participants with the visuospatial and auditory *n-*back simultaneously. The task requires participants to indicate whether the location in which the face is appearing on the current trial is the same as the location in which a face appeared *n* trials back. At the same time, they indicate whether the word they are hearing on the current trial is the same as the word *n*-trials back. The response options include four buttons: “No Match” for non-target trials, “Location Match”, for trials including only a visuospatial target, “Word Match”, for trials including only an auditory target, and “Both Match” for trials including both an auditory and visuospatial target. One third of the target trials are visuospatial targets, one third auditory targets, and one third dual targets.

***Placebo training.*** The P-Training task requires participants to indicate via button press (“Match”, “No Match”) whether two panels display exactly the same stimuli in the same positions on a grid. In the shapes version the stimuli are random geometric shapes. The faces and words versions include the same stimuli as the AffeCT. Each trial is self-timed up to a maximum of 90 s after which participants are asked to respond more quickly. The initial trial includes 5 items per panel, the number of items per panel increases with participant’s performance. 

### Pre- and post-training session assessments

**Figure 3.**
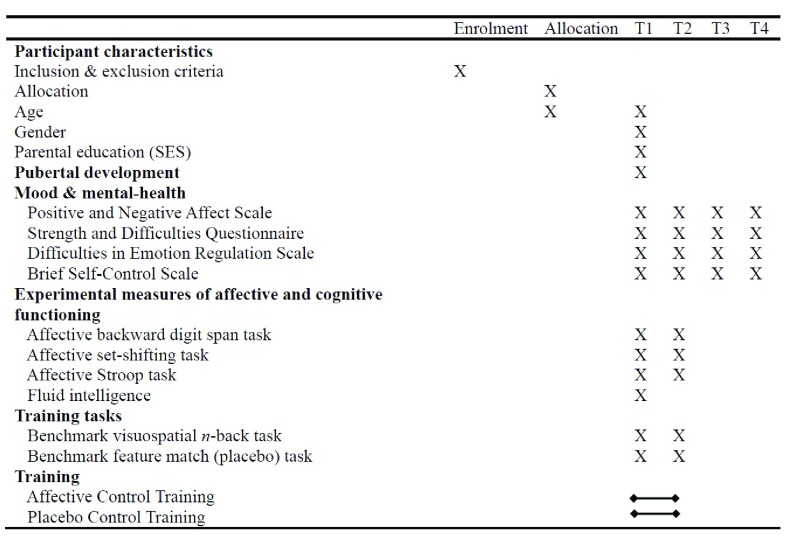
Schedule of enrolment, interventions and assessments (SPIRIT). SPIRIT = Standard Protocol Items: Recommendations for Interventional Trials. T1 = pre-training assessment; T2 = post-training assessment; T3 = 1-month follow-up assessment; T4 = 1-year follow-up assessment.

For an overview of all measures that will be included in T1-T4 see Figure 3.

***Demographics.*** Self-identified gender, ethnicity and parental education level will be assessed. Parental education will be included as a proxy measure for socioeconomic status (SES). Parental education has been shown to be a robust indicator of SES ^58^ and has been previously used by our group in similar samples (e.g., 59).

***Pubertal development.*** Pubertal development will be assessed with the well-validated, self-report Pubertal Development Scale ^60^. The scale will be sent to participants via email link, so that they can complete it in private at home.

***Self-reported mood and mental health.*** All self-report measures will be administered on a computer screen.

*Positive and negative affect.* To assess current positive and negative affect participants will complete the state version of the Positive and Negative Affect Schedule ^61^. The scale requires individuals to rate the extent to which 10 positive and 10 negative adjectives describe them. We will ask participants to rate the adjectives with respect to how well they describe them over the past week.

*Mental health difficulties.* Mental health problems will be assessed with the Strengths and Difficulties questionnaire. The questionnaire is a 25-item self-report measure, which is divided into five subscales ^62, 63^. Four of the subscales measure difficulties and one subscale measures a strength, prosocial behaviour. The difficulties subscales assess emotional symptoms (internalizing symptoms), conduct problems, hyperactivity/inattention and peer relationship problems. The measure has been shown to have good psychometric properties in the age group that will be recruited for the current study (Cronbach’s *α* of 0.80), as well as good sensitivity, specificity and prospective utility ^64– 66^.

*Emotion regulation.* The 36-item Difficulties in Emotion Regulation Scale (DERS) will be administered to assess emotion regulation ^67^. The DERS has six subscales that measure: *non-acceptance*, the propensity to experience secondary negative emotions in response to negative emotions; *goals*, difficulties engaging with goal-directed behaviours when upset; *impulse*, the ability to control one’s behaviour when experiencing negative emotions; *awareness*, the tendency to attend to emotions; *strategies,* individuals’ perception that emotions cannot be controlled; and *clarity*; individuals’ ability to correctly identify their emotions ^67^. The scale has shown high internal consistency, Cronbach’s *α* = 0.93 ^67^ and has been reliably used in the age range included in the current study ^68^.

*Self-regulation.* The Brief Version ^69^ of the Self-Control Scale ^70^ will be administered to measure self-regulation. The scale has 13 items and has shown good internal consistency ^69, 71^, 

***Cognitive-affective task performance.*** Three tasks will be included to assess the impact of AffeCT relative to P-Training on the different facets of affective control: inhibition, updating and set-shifting.

*Inhibition.* Inhibition of affective interference will be assessed using an affective Stroop task ^41^. In this modified version of the task participants indicate whether adjectives are happy or sad. The words are superimposed on the image of a face, resulting in three trial types: congruent (emotions of the word and face are matched), incongruent (emotions of the word and face are mismatched) or neutral (the word is superimposed on scrambled face image). This modified version of the task includes only happy and sad as emotion categories, whereas the original task also included words and faces expressing anger. The current version also includes only four words per emotion category and the faces are from two adult actors and two child/adolescent actors (50% female). These modifications were made to adapt the difficulty level of the task for younger participants and to make the stimuli age-appropriate. The face stimuli are derived from the same face databases as the training stimuli.

Feedback is provided after each trial with a red or green border appearing around the image for 200 ms, indicating an error or correct response, respectively. Trials are self-paced up to 4 s. If no response is detected a red border appears and the next trial is presented. There are 96 trials in total with each actor being paired with each of the eight adjectives in each condition.

*Shifting.* The capacity to shift flexibly between task-demands will be assessed using an affective set-shifting task. The task is an affective version of the Madrid Card Sorting Task ^72^. Participants are dealt a card, which they are asked to assign to one of four decks according to three possible sorting rules: card color, number of items and shape (neutral version) or emotional expression (affective version). Sorting rule switch randomly after 6 to 9 trials (on average after 8 trials). Each rule is presented twice in the neutral and affective versions each, resulting in 96 trials. Participants are required to respond within 30 s, after which the trials are recorded as an error. The presentation order of the affective and neutral versions is randomized. Performance on the task is operationalized as random errors. These are errors that occur on any trial in the series after the initial two trials (needed to establish the correct sorting rule). Random errors are most reliably associated with mental health outcomes in adolescents on this version of the task ^12^.

*Updating.* Updating will be assessed with an affective backward digit span task, where participants are presented with digits (1500 ms) in serial order. The task starts with two digits per trial. Following the final digit in each trial, a keypad appears and participants are required to enter the digits in reverse order. Each span level is presented twice on this task. At least one out of two correct trials per span level is needed for progression to the next level. If both trials are incorrect the task is terminated. To manipulate valence, the digits are presented over negative and neutral background images. The images are from the Geneva Affective Picture Database ^73^.

*Fluid intelligence.* The 12-item version of the Raven’s Advanced Progressive Matrices will be used to assess intelligence ^74^. Participants will be told that they should complete the task as quickly as possible. The measure has good psychometric properties ^75^. We chose the Raven’s Advanced Progressive Matrices because it is sensitive in the wide age range included in the current study.

***Benchmark training tasks.*** The benchmark versions of the training tasks will be identical to the training versions with a few exceptions noted below.

*Visuospatial n-back tasks*. The benchmark version of AffeCT is identical to the visuospatial *n*-back that will be used in the AffeCT with the exception that in the benchmarking version of the task only four blocks will be presented. Two of the blocks will include faces and two blocks will include scrambled faces.

*Placebo task*. For the benchmark version of the P-Training we will present the faces version of the feature match task. The version is identical to the training version with the exception that it is only 90 s long.

### Data management

Following study completion all data will be linked-anonymized, with the linking documents being kept on separate encrypted drives. Fully anonymized data will be made open access through managed open access following the publication of our findings. That is, any researcher will be provided with our data if they consent to adhere to the General Data Protection Regulation and the British Psychological Society’s Code of Ethics and Conduct. Our consent procedure will inform participants of these data storage and sharing procedures.

### Statistical analyses

Statistical analyses will be performed using R ^76^. Prior to all hypotheses testing, the two training groups will be compared on age using a Bayesian *t*-test to ensure that stratification was successful. The groups will then be compared on the following potential confounds using non-parametric Chi-square tests for binary and general linear modelling for continuous variables: gender, parental education, pubertal stage, intelligence, time interval between pre- and post-training (days); testing location and testing groups size to experimenter ratio. Any variables showing significant group differences at baseline will be added to all subsequent group comparisons as covariate.

**Figure 4.**
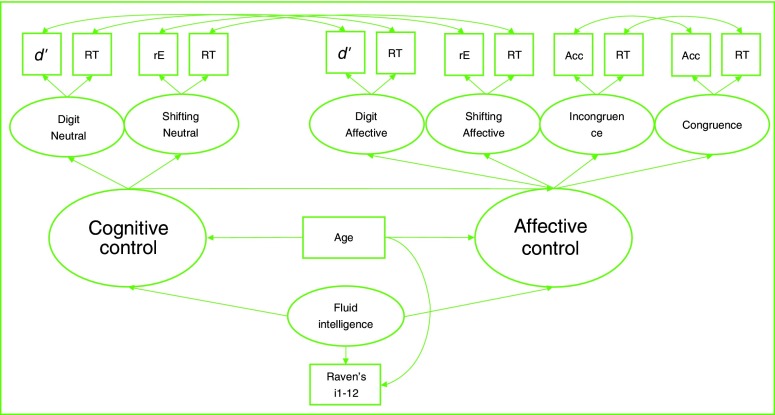
Predicted structure of affective and cognitive functioning at baseline. The figure offers a schematic representation of the predicted structure of cognitive and affective control in adolescents. Raven’s i1 – 12 = items 1 – 12 on the Raven’s Advanced Progressive Matrices; d’ = d prime on backward digit span; RT = reaction time; rE = proportion random errors; Acc = percentage trials correct. Rectangular boxes = measured variables; ovals = latent constructs.

Next, we will explore the structure of our outcome measures of interest at baseline. Specifically, we will explore the structure of affective and cognitive functioning using structural equation modelling (SEM). We hypothesize that cognitive control is best modelled using separate factors for affective, versus neutral, item content, such that the model in Figure 4 will outperform a single-factor model.

To investigate the first hypothesis ( *affective control training hypothesis*) we will use mixed effects modelling with training group as fixed effect and time as within-subject effect. The outcome of interest will be *d*’ achieved on the affective *n*-back task. Additionally, we will explore the impact of training on reaction time as a secondary outcome of interest. For the secondary analyses to be considered significant, we will apply a Bonferroni correction to reduce the threshold for statistical significance for two comparisons (accuracy and reaction) to α ≤ .025. We will then explore whether any effects of group and time are moderated by total training time (mins), total number of individual training sessions and ratio of time spent training on training task C relative to A and B. We plan to include these overlapping measures separately as they arguably provide different types of information. Specifically, total training time will allow us to explore dose-response relationships. The other two measures, we propose, index more motivational factors such as diligence and/or motivation by the monetary incentive. To facilitate interpretation of any potential moderating effect, we will use a SEM trees approach to these moderators and enter the relevant groupings as moderators. We will additionally investigate the effect of time spent on each training version separately on the outcome of interest and compare the effect of time spent on the single versions versus time spent on the dual version.

Our second hypothesis ( *affective control facets hypothesis*), that AffeCT will improve inhibition, updating and shifting, will be tested with a multivariate mixed effects model. Time and group will be included as fixed effects and the three measures of affective control as outcomes of interest (i.e., working memory updating, inhibition, and set-shifting). The primary outcome of interest is accuracy, as this has been shown to be a more sensitive than reaction time in dissociating between individuals with and without mental health problems ^7^. As with the first hypothesis, we will investigate whether any training benefits are moderated by total training time (mins), total number of individual training sessions or time spent training on task C relative to A and B. The analyses will be repeated with reaction time as a secondary outcome of interest.

Our *age-related change hypothesis*, will be tested by including age a moderator in the multivariate mixed effects model used to test the *affective control training hypothesis*. The potentially moderating effect of age will be investigated using SEM trees. SEM trees identify age groupings to the benefits conferred by training. While the exploration is data-driven, it is theoretically informed by the literature showing differential effects of cognitive training on young compared to older adolescents ^34^. A second exploratory analysis will include age as a continuous variable in the same model to investigate any linear or polynomial effects of age.

Fourth, to test the *mental health hypothesis,* we will use latent change score models, a subclass of SEM approaches ^77^, which naturally allows for the integration of predictors of rates of change (e.g. improvements in mental health). Specifically, we will investigate whether pre- to post-training changes in the affective control factor established at baseline are associated with fewer self-reported mental health problems and emotion regulation difficulties, as well as higher levels of self-reported self-control at each assessment time point. The primary analyses will include the post-training and one-month follow-up assessment. Secondary analyses will include the one-year follow-up.

## Research ethics approval

Ethical approval for this study has been conferred by the University College London (UCL) Research Ethics Committee on 23 April 2018; Project ID:12753/002. Any protocol amendments will be submitted to the UCL Research Ethics Committee Chair for approval and recorded on the Open Science Framework pre-registration documentation.

## Consent and assent procedures

Assent and consent will be obtained from prospective participants and their legal guardians, respectively. For participants under 18 years, study information and consent forms will first be sent to the parents. Parents will then have the opportunity to read the information and contact the research team with any potential questions. Children of parents who provide consent will be asked to provide written informed assent. Participants aged 18 years and over will be asked to provide written informed consent. When participants are tested at the pre- and post-training assessments they will be reminded that they can withdraw consent at any time before, during or after the study without any consequence and that they will be compensated for any part of the study completed until withdrawal.

## Availability of data

Consent from participants will be obtained to share data through managed access. Researchers wishing to access the data need to consent to storing and analyzing the data in line with the General Data Protection Regulations and the British Psychological Society’s Code of Ethics and Conduct.

## Dissemination policy

The study has a multi-component dissemination policy: academic, stake-holders and public.

**Academic.** Standard academic dissemination of the study results will be sought through journal publications. Findings will also be communicated at scientific conferences and where permitted by journal regulations published on pre-print archives.

**Stakeholders.** Findings will be communicated via email to all research participants in a newsletter style communication. The main trial outcome paper will also be submitted as a *Frontiers for Young Minds* article and if accepted sent to all participants. We will further present the findings during a school talk in any of the participating schools that are interested in this option.

**Public.** The findings will also be communicated to the public by presenting them at public talks as well as through social media and, if interest can be generated, conventional broadcast or print media.

## Trial status

The trial data collection started 21 September 2018 and the funding end date for this trial is 08 January 2022. Pre-training assessments have been completed in 64 participants, but none of these participants have completed training or any post-training assessments. This is protocol version 1 (30 November 2018).

## Conclusions

If, as predicted, the proposed study finds that AffeCT successfully improves affective control in adolescents, there would be significant potential benefits to adolescent mental health. As a free app, the training would also be scalable and easy to disseminate across a wide range of settings.

## Data availability

### Underlying data

No data are association with this article.

### Extended data

Open Science Framework: Protocol for an App-Based Affective Control Training for Adolescents. https://doi.org/10.17605/OSF.IO/6THSN ^44^

This project contains the following extended data:

- Supplementary Materials - WOR.pdf (Additional methods to main manuscript and completed SPIRIT checklist)

### Reporting guidelines

Open Science Framework: SPIRIT checklist for Protocol for an App-Based Affective Control Training for Adolescents. https://doi.org/10.17605/OSF.IO/6THSN ^44^

Data are available under the terms of the Creative Commons Zero "No rights reserved" data waiver (CC0 1.0 Public domain dedication).
