# Supplementary material for: Protocol for an app-based affective control training for adolescents: proof-of-principle double-blind randomized controlled trial
Source: Wellcome Open Res. 2019 Oct 2;4:91. Originally published 2019 Jun 7. [Version 2] doi: 10.12688/wellcomeopenres.15229.2 (PMC6600858; doi:10.12688/wellcomeopenres.15229.2)
Supplement: Peer review history

# Peer review: Reviewer response for version 2

**DOI:** 10.21956/wellcomeopenres.16951.r36632

**Published:** 2019-11-19

**Authors:** Noga Cohen

**Affiliations:** 1 Department of Special Education, University of Haifa, Haifa, Israel; 2 The Edmond J. Safra Brain Research Center for the Study of Learning Disabilities, University of Haifa, Haifa, Israel

The authors addressed my comments. I am looking forward to seeing the results of this study!

I confirm that I have read this submission and believe that I have an appropriate level of expertise to confirm that it is of an acceptable scientific standard.

# Peer review: Reviewer response for version 1

**DOI:** 10.21956/wellcomeopenres.16620.r35724

**Published:** 2019-07-02

**Authors:** Kristof Hoorelbeke

**Affiliations:** 1 Department of Experimental Clinical and Health Psychology, Ghent University, Ghent, Belgium

The presented design allows to address key questions of which the results will significantly expand our knowledge regarding the potential of cognitive control training as a preventive intervention for internalizing disorders in adolescents. An important advantage of this study is that it allows to explore long-term transfer effects. The manuscript is well-written, provides a clear overview of the design of the study and a transparent description of the to be conducted analyses. As such, I would like to compliment the researchers for the Open Science approach and fully support/endorse this timely study.

 However, some questions remain (at least partially) unaddressed in the current manuscript:

1. The authors allow the participants to decide on which training task they complete following session three while suggesting them to conduct the dual *n*-back version. The rationale for this is not fully clear to me. In addition, this is likely to yield multiple training trajectories reflecting different difficulty levels (e.g., mixed single vs. double back tasks). This may form a confounding factor for some of the presented analyses. In addition, it complicates evaluation of training progress over time.
2. Related to this, the authors state that “opting to engage in a more challenging, but potentially more beneficial task is an index of self-regulation”. From a developmental perspective the authors target a relatively wide age span (i.e., 11 – 19 years), inherently resulting in a sample showing strong heterogeneity in executive functions. As such, multiple other factors are likely to drive this choice (e.g., difficulty of the task, [reduced] maturation of executive control regions [due to a history of internalizing psychopathology], age appropriateness and attractiveness of the selected training procedures for the age group, etc., each of which may interact with other motivational factors). In addition, it would be interesting to also explicitly assess and model factors such as user engagement and task motivation.
3. Moreover, the introduction lacks a clear rationale for the age-related change hypothesis.
4. Although mixed yet encouraging findings have been presented regarding emotional transfer effects of cognitive control interventions, establishing cognitive transfer has been more challenging. In particular, cognitive transfer effects are often found to be task-specific. In a recent meta-analysis, *n*-back tasks have been classified as indicators of updating ability (Zetsche *et al*., (2018) ^1^). To what extent can cognitive transfer effects be expected for other executive control functions such as inhibition and shifting ability, and how does this relate to emotional transfer effects?
5. Throughout the training procedure, the authors reinforce participants to conduct multiple sessions per day. What is the rationale for this, what do the authors consider as the ‘optimal dose’ for this type of cognitive control training and sample?
6. Developing a placebo/control training task is a challenging endeavor. However, the presented “placebo training” seems to be a non-adaptive task and as such does not allow to fully account for motivational effects of undergoing training given that the training procedure is adaptive. The authors could potentially control for this by adding a measure such as the Credibility and Expectancy Questionnaire at baseline and following training.

Sadly, data-collection has already commenced for this study, which limits the modifications that can be made to the design to take into account the concerns raised by the reviewers of the presented protocol.

 Nonetheless, I very much look forward for the results of this interesting study.

I confirm that I have read this submission and believe that I have an appropriate level of expertise to confirm that it is of an acceptable scientific standard.

## References

[1] : Shedding light on the association between repetitive negative thinking and deficits in cognitive control - A meta-analysis. *Clin Psychol Rev*.63: 10.1016/j.cpr.2018.06.001 56-65 10.1016/j.cpr.2018.06.001 29913351

# Response

**Published:** 2019-09-23

**Authors:** Susanne Schweizer

Dear Dr Hoorelbeke,

Thank you for your helpful feedback on our protocol. Please find our responses below to the issues you raised in your report. Your original comments are printed in bold and our responses are copied below. Where appropriate we copied the amended/added sections from the manuscript in italics.

**The authors allow the participants to decide on which training task they complete following session three while suggesting them to conduct the dual n-back version. The rationale for this is not fully clear to me. In addition, this is likely to yield multiple training trajectories reflecting different difficulty levels (e.g., mixed single vs. double back tasks). This may form a confounding factor for some of the presented analyses. In addition, it complicates evaluation of training progress over time.**

**Response: **Please see our response to Dr Cohen.

**Related to this, the authors state that “opting to engage in a more challenging, but potentially more beneficial task is an index of self-regulation”. From a developmental perspective the authors target a relatively wide age span (i.e., 11 – 19 years), inherently resulting in a sample showing strong heterogeneity in executive functions. As such, multiple other factors are likely to drive this choice (e.g., difficulty of the task, [reduced] maturation of executive control regions [due to a history of internalizing psychopathology], age appropriateness and attractiveness of the selected training procedures for the age group, etc., each of which may interact with other motivational factors). In addition, it would be interesting to also explicitly assess and model factors such as user engagement and task motivation.**

**Response: **We agree with the proposed age-related mechanisms, which is why we are explicitly investigating the moderating role of age on training effectiveness.

Unfortunately, we do not have other metrics of task motivation and user engagement than time spent training, which is why we cannot model these factors as proposed by the reviewer.

**Moreover, the introduction lacks a clear rationale for the age-related change hypothesis.**

**Response: **Please see our response to Dr Cohen.

**Although mixed yet encouraging findings have been presented regarding emotional transfer effects of cognitive control interventions, establishing cognitive transfer has been more challenging. In particular, cognitive transfer effects are often found to be task-specific. In a recent meta-analysis, n-back tasks have been classified as indicators of updating ability (Zetsche et al., (2018)1). To what extent can cognitive transfer effects be expected for other executive control functions such as inhibition and shifting ability, and how does this relate to emotional transfer effects?**

**Response: ** This is an important question and one which has not been investigated in the context of affective control, which is why we are exploring the effectiveness of transfer to other facets of affective control. In a previous study we have shown transfer to affective inhibition as measured by the Stroop task included (see our response to Dr Cohen). The effects of the training on affective shifting, however, remain unexplored.

**Throughout the training procedure, the authors reinforce participants to conduct multiple sessions per day. What is the rationale for this, what do the authors consider as the ‘optimal dose’ for this type of cognitive control training and sample?**

**Response: ** Given the relative lack of work in this age group and empirically driven “recommended dose” cannot be determined. As noted in our response to Dr Cohen the rationale for including the option to engage in multiple shorter training session is to maximize potential engagement of this age group with the training.

**Developing a placebo/control training task is a challenging endeavor. However, the presented “placebo training” seems to be a non-adaptive task and as such does not allow to fully account for motivational effects of undergoing training given that the training procedure is adaptive. The authors could potentially control for this by adding a measure such as the Credibility and Expectancy Questionnaire at baseline and following training.**

**Response: **We apologize for omitting this information in the previous version. The placebo training is also adaptive and we now explicitly state this in the training description:

*“The initial trial includes 5 items per panel, the number of items per panel increases with participant’s performance.”*

# Peer review: Reviewer response for version 1

**DOI:** 10.21956/wellcomeopenres.16620.r35725

**Published:** 2019-06-27

**Authors:** Max Owens

**Affiliations:** 1 University of South Florida St. Petersburg (USFSP), St. Petersburg, FL, USA

Summary:

 The proposed protocol outlines a double-blind and app-based training study seeking to improve affective cognitive control in adolescents (11-19 years). Training will consist of online practice on an adaptive and affective version of the *n*-back task, with participants allowed to choose between audio, visual and a dual-audio visual version across 11-days of a 14 day training period. In addition to the training task participants will complete a battery of mood and mental health questionnaires, as well as a battery of cognitive-affective tasks before and after training. The effects of training on changes in mood and mental health and task performance will be compared to a placebo control group completing one of three visual matching tasks consisting of shapes, words or faces. The author’s will be testing four hypotheses predicting training related change in affective control on the *n*-back task, improvement on cognitive-affective tasks relative to the control group, that the benefits of training will decrease with age and that increased training effects on affective control will be associated with lower self-reported mood and mental health problems.

 Critique:

 The protocol is written with a very clear rationale and objectives. The methods are clearly described and seem appropriate for the objectives of the training, however please also include the maximum *n*-back level training participants can achieve. The study design seems largely appropriate as well, and has several strengths, including the use of an active control group, multiple outcome measures and follow-up periods. However, centrally, the reasoning behind allowing participants to choose which version of the training to take could be more clearly supported. If the research question is to compare the effectiveness of affective control training, why have participants potentially only completed less effective versions of the training (i.e. audio and visual only) versus the dual version? Additionally, given the adaptive nature of the tasks, increased improvement would seem to suggest a degree of self-regulation, so this construct could use a clearer operationalization.

I confirm that I have read this submission and believe that I have an appropriate level of expertise to confirm that it is of an acceptable scientific standard.

# Response

**Published:** 2019-09-23

**Authors:** Susanne Schweizer

Dear Dr Owens,

Thank you for your helpful feedback on our protocol. Please find our responses below to the issues you raised in your report. Your original comments are printed in bold and our responses are copied below. Where appropriate we copied the amended/added sections from the manuscript in italics.

**However please also include the maximum n-back level training participants can achieve.**

**Response: **The maximum level of *n *back is not capped.

**However, centrally, the reasoning behind allowing participants to choose which version of the training to take could be more clearly supported. If the research question is to compare the effectiveness of affective control training, why have participants potentially only completed less effective versions of the training (i.e. audio and visual only) versus the dual version?**

**Response: **Please see our response to Dr Cohen.

**Additionally, given the adaptive nature of the tasks, increased improvement would seem to suggest a degree of self-regulation, so this construct could use a clearer operationalization.**

**Response: **The interactive role of improvement with our operationalization of self-regulation is an important consideration, which we will integrate into the interpretation of our results.

# Peer review: Reviewer response for version 1

**DOI:** 10.21956/wellcomeopenres.16620.r35727

**Published:** 2019-06-25

**Authors:** Noga Cohen

**Affiliations:** 1 Department of Special Education, University of Haifa, Haifa, Israel; 2 The Edmond J. Safra Brain Research Center for the Study of Learning Disabilities, University of Haifa, Haifa, Israel

In this report, Schweizer and colleagues propose a protocol for an app-based 14 day training procedure that is predicted to improve affective control, mental health, emotion regulation and self-regulation among youths (11-19 years).

 The authors propose an important and timely study, designed to test whether affective control training can be beneficial for youths. The design proposed in this protocol is based on the authors’ prior work showing improved emotion regulation following an affective control (working memory) training.

 The paper is well written and the proposed study is expected to advance our understanding on the mechanisms involved in emotion regulation and behavioral deficits among youths, as well as open new avenues for treatment. My comments are mainly related to clarification of tasks-related aspects.

 What is the rational for allowing participants to choose the version of the task on days 4-14?

 Moreover, by reading the “The present study” paragraph it is not clear whether participants in the control group (placebo training) will receive the same instructions - allowing them to choose and prompting them to prioritize one of the versions over the other two versions. Although this information is mentioned later in the paper, I suggest referring to this issue already in “the present study” paragraph.

 The authors plan to include both positive and negative stimuli in the affective control training. Are there studies showing beneficial outcomes for this type of training when positive stimuli are used (besides work on eating and addiction with the go/no-go and stop signal tasks)? Can the authors elaborate a bit more about their decision to include positive stimuli in the training?

 Can the authors say something about the similarity/difference of the affective control and placebo training in regard to difficulty? Are accuracy rate and mean reaction time more or less similar in the working memory and perceptual tasks? It can be nice to show that training outcomes are not modulated by task difficulty (therefore strengthening the authors’ notion that the outcomes are specific to improvement in affective control).

 In line with the previous comment, assessing the links between training outcomes in terms of the cognitive/affective control tasks and the self-report measures can be highly valuable to the understanding of the processes by which the training influence emotion regulation, mood, as well as psychological and behavioral difficulties.

 Pre/post changes in inhibition will be assessed using a modified Stroop task. Did the authors consider using a more “classic” inhibition task (e.g., go/no-go, stop signal)? Why Stroop?

 In the predictions section the authors write that they plan to test improvement in affective control by using an affective *n*-back task (which is different from the training task). The authors mention later in the paper that this task will be administered before and after the training. I suggest mentioning this before the “Methods” section because when I read the predictions section it was not clear to me. In addition, why not use the training task itself to assess changes in affective control? Is it because participants choose the version of the task on days 4-14?

 The authors predict that the benefits of AC-Training will decrease with age (age-related change hypothesis). I advise the authors to relate to this issue in the introduction. Is this prediction based on prior literature?

 The authors write that participants will complete 14 days of training within a four-week period. Moreover, they mention that there will be no limit on the number of training sessions participants can complete during a day. Do they mean that during the 4-week period there are 14 specific days in which participants can do the training? On a training day, can participants do a 2-minute task 5 times to complete the session? I find it hard to follow the training procedure. Moreover, what is the rational for enabling participants to choose the number of training sessions?

 On each training session, participants will be asked “Are you trying to change the way you feel right now?” and could choose a strategy - distraction, problem-solving, behavioural activation, reappraisal, avoidance, social support, acceptance or other. I guess that these strategies will be explained at the pre-training session. Can the authors relate to this? They can do that in the “extended data” document if they prefer not to include these details in the main paper.

 What software will be used to program the training apps, questionnaires, and pre/post tasks? Will these apps/tasks be available for other researchers? Are the apps compatible with both Android and Apple smartphone devices?

 The authors mention that “total training time will allow us to explore dose-response relationships”. However, this time may also reflect motivation/choice-related factors as participants choose how much time they wish to spend on the task.

I confirm that I have read this submission and believe that I have an appropriate level of expertise to confirm that it is of an acceptable scientific standard, however I have significant reservations, as outlined above.

# Response

**Published:** 2019-09-23

**Authors:** Susanne Schweizer

Dear Dr Cohen,

Thank you for your helpful feedback on our protocol. Please find our responses below to the issues you raised in your report. Your original comments are printed in bold and our responses are copied below. Where appropriate we copied the amended/added sections from the manuscript in italics.

**What is the rational for allowing participants to choose the version of the task on days 4-14?**

**Response: **We are interested in exploring the role of self-regulation in cognitive training. Providing a rationale for one training (C) being associated with superior benefits compared to A and B should motivate individuals to select C more often. However, C is more cognitively demanding, therefore requiring self-regulation to engage in this task over the others for potential future benefits. We now elaborate on this further in the manuscript.

*“On the first three days of the 14-day training programme, participants will train on one version each day. On days 4–14, participants are free to select any or all of the training versions. However, both training groups will be provided with a rationale suggesting that training on version C is likely to confer more benefits than the other two versions. By providing a rationale for one training being associated with superior benefits compared to others, participants should be motivated to select C over the other two training versions. However, C is more cognitively demanding, therefore requiring self-regulation to engage in this task over the others for potential future benefits. We hypothesise that opting to engage in a more challenging but potentially more beneficial task is an index of self-regulation, which has been shown to be associated with mental health across the lifespan ^36^ . This will allow us to explore the role of self-regulation in cognitive training and any effects on mental health outcomes.”*

**Moreover, by reading the “The present study” paragraph it is not clear whether participants in the control group (placebo training) will receive the same instructions - allowing them to choose and prompting them to prioritize one of the versions over the other two versions. Although this information is mentioned later in the paper, I suggest referring to this issue already in “the present study” paragraph.**

**Response: **We have modified the section accordingly (see above).

**The authors plan to include both positive and negative stimuli in the affective control training. Are there studies showing beneficial outcomes for this type of training when positive stimuli are used (besides work on eating and addiction with the go/no-go and stop signal tasks)? Can the authors elaborate a bit more about their decision to include positive stimuli in the training?**

**Response: **We opted to train affective control across valences because aberrant reward processing is a core characteristic of mood disorders. Similarly, we have recently shown in a meta-analyses that affective control measured with working memory tasks such as the training task is equally impaired in individuals with mental health problems across valences. We now elaborate on this further in the background section:

*“The rationale for including stimuli of different valences is that mental health problems can be characterised by difficulties disengaging from negative material ^37^ , avoidance of negative (e.g., threatening) information ^38^ or aberrant processing of positive information ^39^ . Moreover, we have recently shown in a meta-analysis that, in individuals with mental health problems, affective control, measured with working memory tasks such as the training task, is similar across positive and negative valence ^7^.”*

**Can the authors say something about the similarity/difference of the affective control and placebo training in regard to difficulty? Are accuracy rate and mean reaction time more or less similar in the working memory and perceptual tasks? It can be nice to show that training outcomes are not modulated by task difficulty (therefore strengthening the authors’ notion that the outcomes are specific to improvement in affective control).**

**Response: **While the placebo task is engaging and challenging it cannot be directly compared to affective control training task in terms of either accuracy or reaction time as the former is a self-paced search task. We will consider the potentially confounding effect of task difficulty in our interpretation of our findings.

**In line with the previous comment, assessing the links between training outcomes in terms of the cognitive/affective control tasks and the self-report measures can be highly valuable to the understanding of the processes by which the training influence emotion regulation, mood, as well as psychological and behavioral difficulties.** 

**Pre/post changes in inhibition will be assessed using a modified Stroop task. Did the authors consider using a more “classic” inhibition task (e.g., go/no-go, stop signal)? Why Stroop?**

**Response: **We have included the Stroop task to replicate findings from the adult literature, which have shown the affective control performance to improve affective Stroop performance (Schweizer et al., *PLoS ONE*, 2011).

**In the predictions section the authors write that they plan to test improvement in affective control by using an affective n-back task (which is different from the training task). The authors mention later in the paper that this task will be administered before and after the training. I suggest mentioning this before the “Methods” section because when I read the predictions section it was not clear to me. In addition, why not use the training task itself to assess changes in affective control? Is it because participants choose the version of the task on days 4-14?**

**Response: **We have removed the reference to the different versions as we appreciate the confusion this caused. As noted in the methods, the task is the same but includes non-trained stimuli sets as well as a different progression and termination rule. That is, each level of *n *is presented twice. The task terminates once both blocks at a given level are completed incorrectly.

**The authors predict that the benefits of AC-Training will decrease with age (age-related change hypothesis). I advise the authors to relate to this issue in the introduction. Is this prediction based on prior literature?**

**Reponses: **We have modified the hypothesis to be non-directional as we agree that there is not sufficient evidence to support a directional hypothesis. We now also refer the age-related hypothesis earlier stating:

*“Specifically, we will explore whether AffeCT improves adolescents’ mental health and emotion regulation capacity and whether the magnitude of these improvements differs as a function of age.*

*To investigate the potential of AffeCT in adolescents, the current study will include 200 adolescents (11-19 years). Including this age range will allow us to investigate the potential age-related differences in the effectiveness of training, as shown in studies using cool cognitive training paradigms ^76^ .”*

**The authors write that participants will complete 14 days of training within a four-week period. Moreover, they mention that there will be no limit on the number of training sessions participants can complete during a day. Do they mean that during the 4-week period there are 14 specific days in which participants can do the training? On a training day, can participants do a 2-minute task 5 times to complete the session? I find it hard to follow the training procedure. Moreover, what is the rational for enabling participants to choose the number of training sessions?**

**Response: **A full training regime is considered 14 days but participants can opt to train for more days. There will be no limit on the number of training sessions they can complete during a day. A full training session takes between 20-30 minutes but participants have the option to end the training after 10 minutes. Anything less than 10 minutes will not be counted as a training session. The reason for allowing participants to stop the training after 10 minutes is to increase engagement with the training in this age group. Allowing for multiple training sessions in a day enhances the possibility of maximizing training time in a sample who is likely not to engage in an activity for extended amounts of time but who might be motivated by the monetary compensation to engage in a task repeatedly.

In the methods we note:

*“[…] participants will be given the option to end the training any time from 10 mins onward. The full training session will take between 20–30 mins depending on the level achieved. There will be no limit on the number of training sessions they can complete during a day. Training sessions that are less than 10 mins will not be considered as full training sessions, and will not be included in the analyses, nor will participants be compensated for these sessions.”*

**On each training session, participants will be asked “Are you trying to change the way you feel right now?” and could choose a strategy - distraction, problem-solving, behavioural activation, reappraisal, avoidance, social support, acceptance or other. I guess that these strategies will be explained at the pre-training session. Can the authors relate to this? They can do that in the “extended data” document if they prefer not to include these details in the main paper.**

**Response: **Each strategy is described in the answer options. The strategy names in brackets are not included in the drop down menu.

Are you trying to change the way you feel right now?

No

Yes, by distracting myself. (Distraction)

Yes, by dealing with my problem. (Problem-solving)

Yes, by doing something fun. (Behavioural activation)

Yes, by thinking about the problem differently. (Reappraisal)

Yes, by avoiding the problem. (Avoidance)

Yes, by spending time with family/friends. (Social support)

Yes, by accepting the situation. (Acceptance)

Other

**What software will be used to program the training apps, questionnaires, and pre/post tasks? Will these apps/tasks be available for other researchers? Are the apps compatible with both Android and Apple smartphone devices?**

**Response: **Once the results from this study and other ongoing studies using the apps are published the apps will be made available to other researchers. The apps are compatible with both Android and Apple devices.The app and tasks are coded in javascript and HTML5. To get them to run on the mobile devices we are using a software package called Cordova which converts the Javascript and HTML5 into the native languages of the devices.

**The authors mention that “total training time will allow us to explore dose-response relationships”. However, this time may also reflect motivation/choice-related factors as participants choose how much time they wish to spend on the task.**

**Response: **This is an important consideration, which we will incorporate into the interpretation of our results.

# Peer review: Reviewer response for version 1

**DOI:** 10.21956/wellcomeopenres.16620.r35726

**Published:** 2019-06-25

**Authors:** Nazanin Derakshan

**Affiliations:** 1 Department of Psychological Sciences, Birkbeck, University of London, London, UK

This is an excellent and timely study with much potential for impact on adolescent mental health. The aim is to improve affective control and hopefully emotion regulation strategies in young and older adolescents using affective control training, a paradigm used by the first author before with good results. 

 The study is longitudinal in nature which is to be commended. The longitudinal effects of AC training will be studied on self-report measures of mood and emotional vulnerability and emotion regulation as well as performance measures of executive control functioning and working memory performance, which are crucial to investigate. 

 The methods of analysis addressing each of the predictions are sound and adequate, clearly explained and solid. 

 The characteristics of the AC training and the Placebo (P) training are well defined and easy to follow. I have a few comments/remarks that the authors might want to consider:

 First, would there be a problem in letting the participants 'choose' the single (auditory or visual) or the dual *n*-back training after day 3? I would assume that the single *n*-back can be less demanding on working memory resources than the dual *n*-back which can be more engaging for the participant. In this way, how can we control for possible confounds as a matter of choice of training? How can we compare the efficacy of the single to the dual, which I am guessing is a noteworthy comparison to do anyway, but won't be possible given the unsystematic manipulation if any.

 Second, it isn't clear if participants will do the training at home or at school if they are using an app? There is a mention of an app and that the study will run in schools - in form of a group testing session? Or individually?

 I fully support this study and I hope that the findings can pave the way towards promoting as well as sustaining better mental health in a population in most need of it.

I confirm that I have read this submission and believe that I have an appropriate level of expertise to confirm that it is of an acceptable scientific standard.

# Response

**Published:** 2019-09-23

**Authors:** Susanne Schweizer

Dear Professor Derakshan,

Thank you for your helpful feedback on our protocol. Please find our responses below to the issues you raised in your report. Your original comments are printed in bold and our responses are copied below. Where appropriate we copied the amended/added sections from the manuscript in italics.

**First, would there be a problem in letting the participants 'choose' the single (auditory or visual) or the dual n-back training after day 3? I would assume that the single n-back can be less demanding on working memory resources than the dual n-back which can be more engaging for the participant. In this way, how can we control for possible confounds as a matter of choice of training? How can we compare the efficacy of the single to the dual, which I am guessing is a noteworthy comparison to do anyway, but won't be possible given the unsystematic manipulation if any.**

**Response: **We agree that the effects of the training are likely to vary as a function of the training version selected. As suggested by Professor Derakshan we will look at the effectiveness of training as a function of the training version selected. Specifically, we will look at the effects of time trained on each version on our outcomes of interest. We will additionally also run a comparison of time trained on the dual version versus time trained on the single versions. We now explicitly state this in the analysis section:

*“We will additionally investigate the effect of time spent on each training version separately on the outcome of interest and compare the effect of time spent on the single versions versus time spent on the dual version.”*

**Second, it isn't clear if participants will do the training at home or at school if they are using an app? There is a mention of an app and that the study will run in schools - in form of a group testing session? Or individually?**

**Response: **Participants will do the training in their own time, on their own devices (any device that supports mobile apps). We now specified this further in the methods.

*“Participants complete a pre-training assessment, followed by 14 days of training within a four-week period. The training will be completed by the participants on their own devices (any device that supports mobile apps) outside of school time.”*
